# Database of surface water diversion sites and daily withdrawals for the Upper Colorado River Basin, 1980–2022

**DOI:** 10.1038/s41597-024-04123-0

**Published:** 2024-11-21

**Authors:** Samuel F. Lopez, Jacob E. Knight, Fred D. Tilman, Melissa D. Masbruch, Daniel R. Wise, Casey J. Jones, Matthew P. Miller

**Affiliations:** 1 U.S. Geological Survey, 2329 W Orton Circle, Salt Lake City, UT 84119 USA; 2 U.S. Geological Survey, 520 N. Park Avenue, Tucson, AZ 85719 USA; 3 U.S. Geological Survey, 601 SW 2nd Ave Suite 1950, Portland, OR 97204 USA; 4 U.S. Geological Survey, 720 Gracern Road Suite 129, Columbia, SC 29210 USA; 5 U.S. Geological Survey, 3215 Marine St, Boulder, CO 80303 USA

**Keywords:** Hydrology, Water resources

## Abstract

The Colorado River drains about 8% of the conterminous United States, provides water for 40 million people, and is one of the most overallocated rivers in the world. As the upper Colorado River Basin (UCOL) contributes an estimated 92% of the total basin natural streamflow, knowledge of the location and amount of surface water withdrawals in the UCOL is important for managing the Colorado River system. Since the UCOL encompasses portions of five states, water use data are dispersed among numerous federal and state agency databases, and there is no centralized dataset that documents surface water use within the entire UCOL at a fine spatial and temporal resolution. This article presents an inventory of 1,358 major structures that divert surface water from and within the UCOL with corresponding daily time series withdrawal records from 1980 through 2022. Data compilation efforts, processing methods, and contents of this diversion database are documented, and summary information is provided.

## Background & Summary

The Colorado River is an important water resource in the southwestern United States, supplying drinking water for 40 million people in the region, and water for irrigation of 2.2 million hectares of land^1^. The Colorado River and its tributaries are an essential source of water for at least 22 federally recognized Tribes, 7 National Wildlife Refuges, 4 National Recreation Areas, and 11 National Parks^1^. The Colorado River Basin drains parts of 7 U.S. States and Mexico and is divided into upper and lower basins, as defined by the Colorado River Compact, at Lees Ferry, Arizona, located 1.6 kilometers (km) downstream of the mouth of the Paria River^2^. The flow of the Colorado River in the lower basin is mostly controlled by releases from Glen Canyon Dam, and about 92% of the natural streamflow in the lower river originates in the upper basin^3^. The Colorado River also has been identified as one of the most regulated and overallocated rivers in the world^4^. Water withdrawals throughout the Colorado River Basin are primarily from surface water, making up 78% of total withdrawals from 1985–2010^5^. However, this fraction is notably higher in the Upper Colorado River Basin (UCOL) where surface water diversions made up 98% of total withdrawals over the same period^5^.

The UCOL Integrated Water Availability Assessment (IWAA) project was created by the U.S. Geological Survey (USGS) to evaluate water quantity and quality in both surface and groundwater in the basin, as related to human and ecosystem needs, and as affected by human and natural influences. An important component of the UCOL IWAA project is the development of a groundwater-surface-water hydrologic model of the basin for 1980–2022 to support improved understanding of the relationship between groundwater and surface water in the UCOL and Little Colorado River Basin, investigate how that relationship has changed, and project how it may change in the future as a result of human use of water and climate change. Detailed spatial and temporal information on the diversion of surface water for use elsewhere in the basin, or removal from the basin, is crucial for hydrologic models of the region.

The majority of consumptive water use within the UCOL is associated with local irrigation, with smaller portions also utilized for public and industrial use applications^5^. Additionally, accounting for transfers of surface water within or exports of surface water from the UCOL is an important component of the basin’s water budget. Time series withdrawal records for surface water diversion structures exist in numerous federal, state, and local databases, but the lack of centralized withdrawal records, particularly between different states, creates difficulties in compiling regional water use data. In turn, this creates challenges for researchers, modelers, and water use planners addressing questions related to water availability throughout the UCOL and more generally throughout the U.S.^6^. In recent years, significant efforts have been undertaken to inventory interbasin transfers (water conveyance structures that cross basin boundaries) throughout North America^7–10^, and have contributed to addressing this critical knowledge gap. Further characterization of other diversion structures (e.g., local irrigation canals), in addition to interbasin transfers, will greatly aid in assessments of water resources and water availability, particularly in regions facing water stress such as the UCOL.

**Fig. 1:**
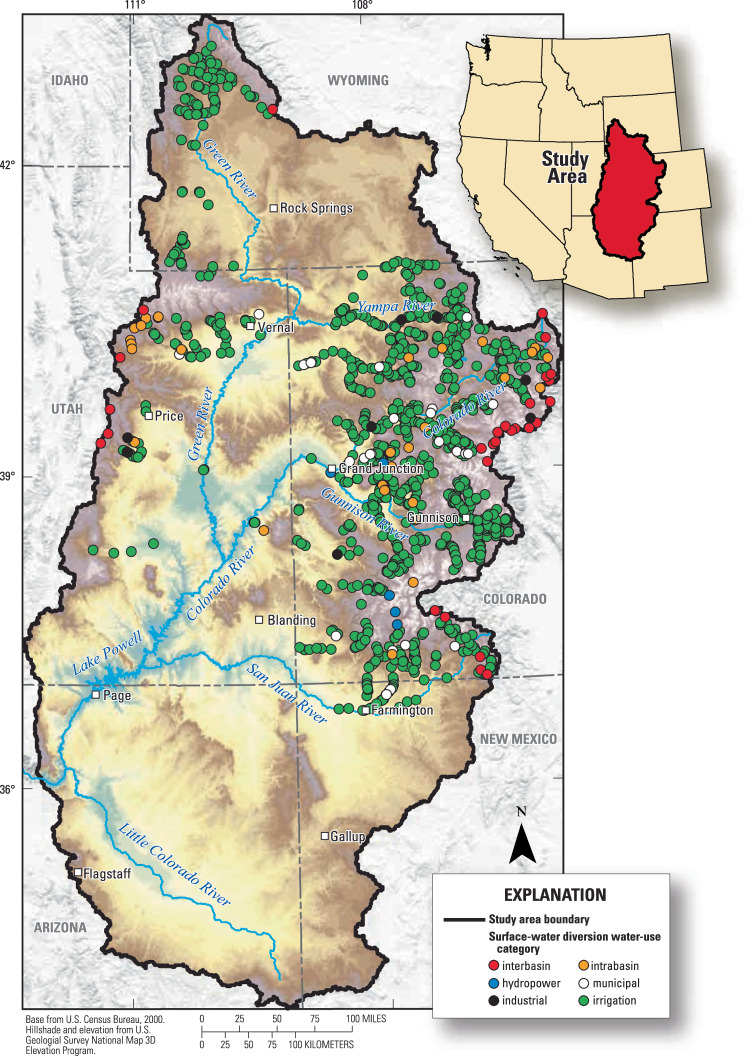
Map of diversion structures with associated time series records by primary use type.

This article presents a database^11^ containing location, water use type, and other relevant information for 1,358 major diversion structures (e.g., canals, pipelines) spanning multiple states within the UCOL and Little Colorado River Basin that were determined to have the capacity to withdraw water at rates greater than 10 cubic feet per second (cfs), the majority of which are irrigation diversions (Fig. 1). Corresponding measured and estimated daily time series withdrawal records from 1980 through 2022 are also presented, including Python^12^ scripts that were developed to retrieve, process, and harmonize data records from several state and federal databases. Also included is site information for other diversion structures with no associated time series withdrawal records that are likely able to divert surface water at rates greater than 10 cfs. All data compilation efforts, processing steps, and contents of this surface water diversion database are provided below.

## Methods

Development of the UCOL surface water diversion database consisted of multiple steps, including (1) collecting and compiling diversion location and time series withdrawal data from the UCOL and Little Colorado River Basin states (Arizona, Colorado, New Mexico, Utah, Wyoming), (2) investigating and assigning a primary water-use category to each diversion structure, (3) estimating daily time series records from spot check and monthly data as necessary, (4) removing outliers, (5) estimating daily time series records for years with no available data, and (6) processing time series withdrawal data from different sources into a consistent format (daily withdrawal rates in cfs). Detailed information for each of these steps is provided in the following sections.

### Data compilation

The dataset described herein consists of surface water diversion locations, time series records of daily withdrawal rates, and water use types for individual diversion structures within the UCOL. Diversion structures with the ability to divert at rates greater than or equal to 10 cfs (0.283 m^3^/s) are included in this dataset. Conveyance capacity of 10 cfs was chosen as a threshold for substantial impacts on water budget components. Since the UCOL includes portions of five different states, code was written in the Python programming language^12^ (see Code Availability) to retrieve, process, filter, and harmonize diversion records from state and federal (USGS and U.S. Bureau of Reclamation [USBR]) data portals, as explained in the state-by-state compilation details that follow. Records for 1,358 diversion structures for the period of 1980 through the end of water year 2022 were available from state and federal sources. The scripts provided allow for compilation of diversion data for other periods of interest or for more recent time periods. Other diversion structures for which data were not available, but which may be of importance owing to their size, are provided for reference (see Data Records section). Specific data compilation methods for Colorado, Wyoming, Utah, New Mexico, and Arizona diversions within the UCOL and Little Colorado River Basin are described below.

Coordinates associated with each diversion location were checked and manually updated as needed to best represent the physical point of diversion. At the time of data compilation, diversion structure location information was not available for all sites. When location information was not available, the physical point of diversion was identified through a combination of maps included in commissioner reports, the USGS National Hydrography Dataset (NHD)^13^, and satellite imagery^14^.

#### Colorado

1,107 of the 1,358 surface water diversion structures contained in this database with associated time series withdrawal records were from locations in Colorado. This accounts for nearly 80% of the total diversion structures in this database, which is generally consistent with the majority ( > 50%) of agricultural lands in the UCOL occurring within the state of Colorado^15^. Colorado diversion records were retrieved from the Colorado Decision Support Systems (CDSS) Structures web portal (https://dwr.state.co.us/Tools/Structures)^16^, a water management system developed by the Colorado Water Conservation Board and the Colorado Division of Water Resources. However, as the CDSS Structures portal has information for 69,713 structures within the San Juan/Dolores, White/Yampa, Colorado, and Gunnison Divisions at the time of writing, this list needed subsetting to include only diversions, and only those determined to have an ability to withdraw at rates greater than or equal to 10 cfs. The State of Colorado maintains Water Resources Planning Models (water allocation models) for all Colorado Water Divisions, and lists of surface water diversions (which provide site name, site identification [ID] number, structure capacity, and associated irrigated acreage) are provided in user’s manuals for each model^17^. These diversion site lists for the San Juan/Dolores, White/Yampa, Colorado, and Gunnison Divisions were combined and subsetted to only include diversion structures with a capacity of 10 cfs (0.283 m^3^/s) or greater.

Modifications to the subsetted CDSS dataset included: 1) the removal of sites associated with laterals (defined here as a canal that diverts water from another canal rather than the original water source) to avoid double counting diverted water; 2) combining nearby sites with limited records or swapping site IDs to achieve the best possible continuous record for a diversion location; and 3) by inspection, adjusting published locations of diversions where necessary to best reflect the likely actual location at which water was removed from the source waterbody. Code was developed to automate retrieval of daily time series diversion data utilizing CDSS web services.

Two large interbasin transfers, the Boustead and Moffat Tunnels, are fed by complex networks of smaller diversions. Both the Boustead and Moffat Tunnels have associated daily time series withdrawal records that represent total amounts of water exported out of the UCOL by these structures. The relative contributions of the smaller diversions that feed the larger tunnels could not be estimated with a high degree of certainty, as these smaller diversion structures lacked robust daily time series withdrawal records. Because of this, time series data are provided for the total amounts of water exported by the Boustead and Moffat Tunnels, but not for the smaller diversions feeding these tunnels. However, structure names, ID numbers, and locations of these smaller diversions are provided in a separate table (see Data Records section).

#### Wyoming

Second to the number of sites in Colorado, 122 of the 1358 surface water diversion structures contained in this database with associated time series withdrawal records were from locations in Wyoming. Publicly available, daily diversion records are housed on the Wyoming State Engineer’s Office (WY SEO) Data Portal (https://seoflow.wyo.gov/)^18^. Daily time series records are available on the portal since ~2010 but are not available for all points of diversion in the state. Additional surface water diversion data dating back to 1980 were obtained from the Wyoming Water Resources Data System upon request in 2021. These data are sourced from USGS gaging station records and State Hydrographer’s records, and contain a mix of daily time series data and spot check records (that is, individual measurements made during site visits and recorded in State Hydrographer reports).

An inventory of major irrigation diversions (Irrigation Diversion Operation and Description) published as a Technical Memorandum for the Wyoming Green River Basin plan^19^ and a similar inventory of industrial water use^20^ were used to identify diversions with permitted flows greater than the defined threshold for this study (10 cfs or greater). Some diversion structures had permitted flows of greater than 10 cfs but lacked publicly available time series withdrawal records. These diversions are listed in a table of non-included, but likely important, UCOL diversions (see Data Records section).

The location of each diversion structure in the Wyoming portion of the UCOL was identified using satellite imagery^14^ and the legal point of diversion (provided as Public Land Survey System coordinates) on the WY SEO permitting portal (https://seoweb.wyo.gov/e-Permit/common/login.aspx)^21^. Provided code automates retrieval of diversion records from the WY SEO data portal and combines with offline records.

#### Utah

101 of the 1,358 surface water diversion structures contained in this database with associated time series withdrawal records were from locations in Utah. Most of the publicly available Utah diversion records are hosted on the Utah Division of Water Rights’ (UTDWR) Water Distribution/Regulation data portal (https://www.waterrights.utah.gov/distinfo/distribution_systems.asp)^22^, and are grouped by distribution system (e.g., watershed). Most records comprise daily time series data, though several diversions were found to have missing records for certain years. Many of the missing data were found in scanned copies of commissioner reports (available by request through the Water Distribution/Regulation portal) that had not yet been transcribed. Monthly diversion data were manually transcribed from these reports and used to estimate daily averages for periods of missing records, as described below.

In addition to diversion data obtained through the UTDWR data portal, three interbasin diversions (the Ephraim, Fairview, and Spring City Tunnels) are associated with USGS streamgages, and records for these diversions were obtained from from USGS’s National Water Inventory System database (NWIS) (10.5066/F7P55KJN)^23^. Records associated with a municipal diversion located in Eastern Utah, the Tyzack Diversion, were obtained from the United States Bureau of Reclamation (USBR) Hydrodata Portal (https://www.usbr.gov/uc/water/hydrodata/)^24^. Additional records were provided by the Central Utah Water Conservancy District (CUWCD) for diversions associated with the Central Utah Project upon request in 2021. Provided code automates retrieval of diversion records from UTDWR, NWIS, and USBR, and appends to offline records obtained from Utah commissioner reports^22^ and CUWCD. In cases where daily records from multiple agencies overlapped, values from the more complete time series dataset were used.

Because of limited structure capacity information, Utah diversions were assessed in a different manner from Colorado or Wyoming. If reported daily flow, or calculated daily average flow from reported monthly totals, reached or exceeded 10 cfs at any time from 1980–2022 at any site, the diversion was considered able to convey flows of at least 10 cfs and the data were included in this project dataset.

No retrievable diversion records were found for three irrigation canals that withdraw water from the Fremont River near Loa, Utah. These diversions are listed in the table of non-included but likely important UCOL diversions (see Data Records section).

#### New Mexico

28 of the 1,358 surface water diversion structures contained in this database with associated time series withdrawal records were from locations in New Mexico. Most publicly available New Mexico diversion records are served on the New Mexico Office of the State Engineer’s Real-Time Water Measurement Information System data portal (http://meas.ose.state.nm.us/)^25^. Daily time series records from the portal are available from 2012 onwards, but additional records dating to 2005 were obtained from the New Mexico Office of the State Engineer (NM OSE) through a data request in 2021. Prior records of surface water diversions are sparse. A New Mexico diversion inventory and/or lists of diversion capacities were not found, so diversions were assessed in a similar manner to Utah. If average daily flow exceeded 10 cfs at any time from 1980–2022, the diversion was considered able to convey flows of at least 10 cfs, and the site was included in the database. Provided code automates retrieval of diversion records from the NM OSE data portal and combines with offline records. Coordinates associated with each diversion location were updated where needed to reflect the physical point of diversion rather than the location of the streamgage used for monitoring.

In addition to diversion data available from the NM OSE data portal, two large New Mexico diversions, Hogback Canal and Navajo Indian Irrigation Project, are monitored by the USGS and USBR, respectively. The codedeveloped for New Mexico data retrieval also automates retrieval of daily time series records for these diversions from the USGS NWIS database^23^ and USBR HydroData portal^24^.

No publicly available diversion records were found for Fruitland Canal, a large irrigation canal that diverts water from the San Juan River west of Farmington, New Mexico. This diversion is listed in the table of non-included, but likely important, UCOL diversions (see Data Records section).

#### Arizona

No daily withdrawal time series records are available for diversion structures in Arizona that are within the study area. Diversion locations were found through the Arizona Department of Water Resources point of diversion dataset (https://app.azwater.gov/querycenter/query.aspx)^26^. The shapefile associated with the point of diversion dataset was clipped to the study area boundary, and subsequently filtered to include points of diversion with water rights above the threshold value. The resulting sites are included in the table of likely important diversions without time series records (see Data Records section), along with their location, water right ID, and water right amount in cfs.

### Assigning water use categories

Each diversion structure was assigned a single water use category based on the primary use of diverted water. Six water use categories were used for characterizing diversions within this dataset, including 1) irrigation, 2) municipal, 3) hydropower, 4) intrabasin, 5) interbasin, and 6) industrial. Definitions of the six use types are provided in Table 1.

**Table 1:** Definitions of water use types for UCOL surface water diversion structures.

Use Type	Definition
Irrigation	Water diverted for irrigation of local croplands.
Municipal	Water diverted for local municipal use.
Industrial	Water diverted for local industrial applications such as mining and coal fired powerplants.
Hydropower	Water diverted for generation of hydropower (non-consumptive). Hydropower diversions were only included if they conveyed water a significant distance from the original point of diversion. On-stream hydropower generation sites (e.g., dams) are not included.
Intrabasin	Water transferred within the UCOL from one waterbody (e.g., stream or reservoir) to another
Interbasin	Water exported from and used outside of the UCOL.

For Colorado diversions, use type was determined from Water Resources Planning Model user’s manuals^17^, which provide lists of individual surface water diversions and use information. Generally, if a diversion was linked to irrigated acreage, irrigation was assigned as the primary use. For non-irrigation diversions, additional information about specific use was provided, which was used to match diversion structures to one of the five other use types. While the majority (93%) of Colorado diversion structures with associated time series records included in this database are irrigation diversions, Colorado contained more intrabasin and interbasin transfers than all other states (Table 2).

**Table 2:** Number of surface water diversion structures with associated time series withdrawal records by state and by use type.

Use Type	Colorado	New Mexico	Utah	Wyoming	Total
Irrigation	1034	26	79	121	1259
Municipal	21	2	2	0	25
Industrial	4	0	3	0	7
Hydropower	5	0	0	0	5
Intrabasin	20	0	12	0	32
Interbasin	23	0	5	1	29
All types	1107	28	101	122	1358

For Wyoming diversions, use type was determined from State Engineer records and technical memoranda associated with the Wyoming Green River Basin Plan^19,20^. Irrigation was identified as the primary use type for all but one of the Wyoming diversions with associated time series records included in this database (Table 2).

For New Mexico diversions, use type was determined from information provided by the State Engineer’s office. Irrigation was identified as the primary use type for almost all New Mexico diversions with associated time series records included in this database (Table 2).

Due to a lack of available information, use type was difficult to determine for some Utah diversions. Use type was estimated via a combination of information contained in commissioner reports, discussions with CUWCD personnel, and interpretation using the NHD^13^ and satellite imagery^14^. While the majority of Utah diversion structures are irrigation diversions, Utah contains several large intrabasin and interbasin transfers associated with large water projects, including the Central Utah Project (Table 2).

### Data processing

All diversion records contained in the database are presented as daily time series data in cfs. However, for many diversion sites, records consisted of spot check measurements or were unavailable for certain years throughout the study period. The following workflow was developed to address the processing of spot check records and estimating diversion rates in years without available records.

#### Spot check records

Most sites with spot check records occur within the state of Wyoming, and these records were processed according to previously established methods^19^. To convert spot check records to daily time series data, linear interpolation was done between spot check flow measurements. If two data points were more than 90 days apart, no interpolation between these points was done to prevent interpolation over periods where structures were not actively diverting (e.g., winter months, where irrigation diversions are typically not active). Unless otherwise stated in the record, it was assumed that the first spot check measurement after a period of inactivity was the date that the canal headgate was turned on. By using these assumptions, estimated amounts may underrepresent diversions at the beginning of the irrigation season (e.g., if canals were turned on prior to site visits/spot check measurements).

#### Monthly records

Monthly totals (in cfs-days) for some Utah sites were obtained from commissioner reports when automated retrieval of daily time series withdrawal records was not possible, and/or when daily records were not available but monthly records were available. To process these data, monthly totals were divided by the number of days per month to estimate withdrawal rates in cfs for each day within a specific month.

#### Filtering

Filtering steps were applied to remove errors and potential outliers from time series data. First, all negative discharge values associated with any diversions were re-assigned a value of zero, as it was assumed that negative discharge values were errors associated with periods of diversion inactivity.

Next, time series outliers for each site were identified and removed using an initial and secondary filter. An initial filter was designed to remove potential outliers from the calculation of the standard deviation of the annual maximum rate. This filter rejected annual maximum rates that were both at least 25 cfs and 2 times greater than the median annual maximum rate. Annual maximum rates from the remaining values were then used to calculate a standard deviation, and the secondary filter was applied to reject any daily values greater than three standard deviations above the median annual maximum rate.

#### Years with no available records

After all spot check records were converted to daily time series data and outliers/errors were removed, monthly average flows for each diversion structure were calculated from all available records throughout the study period. These monthly averages were then utilized to estimate daily withdrawal values that were applied across years without diversion data. For diversions with years within the 1980–2022 study period lacking records, commissioner reports, state engineer documentation, and other sources were checked to assess whether diversions were inactive/non-operational. It was assumed that diversion structures were active in years lacking records, unless otherwise stated in state engineer or other documentation.

## Data Records

The UCOL diversions database is published as a ScienceBase Data Release^11^ and has been assigned a unique digital object identifier (DOI) (10.5066/P1496VHX). Files associated with the dataset are stored in three folders: “input,” “scripts,” and “processed.”

The “input” folder contains a Master Table of diversion structure information stored as a comma separated values file (CSV, ucrb_diversion_master_table). Each diversion structure in the master table is assigned a unique, dataset specific identification number (shortID) and structure name (siteName). A long identifier (siteID), in the format “div_X_Y” where X is the shortID and Y is the use type, directly links each diversion structure to associated time series records. All variables in the Master Table are defined in Table 3. The “input” folder also contains a CSV table of information for diversion structures that lacked associated available time series withdrawal records (“ucrb_diversions_no_records.csv”). All variables in this table are defined in Table 4.

**Table 3:** Features and descriptions for Master Table of UCOL diversion structures with available time series records.

Table Feature	Feature Description
shortID	A unique, database-specific identification number for each diversion structure.
siteID	A unique, database-specific identifier for each diversion structure that links each structure to associated time series records. The format incorporates both the shortID and siteUse for each structure, and is div_X_Y, where X is the shortID and Y is siteUse. Example: div_0045_interbasin
basin	A three-letter basin code for which subbasin within the UCOL the diversion structure is located: lcr = Little Colorado River Basin, grb = Green River Basin, sjb = San Juan River Basin), cms = Colorado Main Stem (Colorado River and Tributaries outside of the Green, San Juan, and Little Colorado River Basins).
state	A two-letter state code for where the diversion structure is located (az = Arizona, co = Colorado, wy = Wyoming, ut = Utah, nm = New Mexico).
siteName	The site name for each diversion structure within the database that contains state and basin information. Example: grb_ut_vat_diversion
siteUse	The primary water use classification for each diversion structure.
destinationCode	Field specific to the USGS groundwater-surface-water hydrologic model. Values either correspond to model subbasin identification numbers where water for irrigation is delivered, or model stream segment identification numbers where water is transferred.
destinationFlag	Field specific to the USGS groundwater-surface-water hydrologic model. A value of −1 denotes water exported from the model area.
dataSource	The data source the Python script accesses for retrieval of online records.
usbrID	The USBR identification number used to automate retrieval of associated daily time series withdrawal records.
usgsID	The USGS identification number used to automate retrieval of associated daily time series withdrawal records.
cdssID	The CDSS identification number used to automate retrieval of associated daily time series withdrawal records.
wyseoID	The WY SEO identification number used to automate retrieval of associated daily time series withdrawal records.
utdwrID	The UTDWR identification number used to automate retrieval of associated daily time series withdrawal records.
nmoseID	The NM OSE identification number used to automate retrieval of associated daily time series withdrawal records.
historicalRecord	Denotes whether the diversion has any historical records (these are contained in the “inputs” folder).
decLat	The latitude of the point of diversion associated with each diversion structure.
decLong	The longitude of the point of diversion associated with each diversion structure.
dest_decLat	The latitude of the discrete point of return flows associated with each diversion structure, if applicable
dest_decLong	The longitude of the discrete point of return flows associated with each diversion structure, if applicable
notes	Notes on the diversion structure, if additional/relevant information was available.
startDate	The date that the first surface water withdrawals at each site commenced. If no information was found (or the date was prior to 1/1/1980), no startDate was assigned.
endDate	The date that surface water withdrawals ended and the site became inactive. If no information was found, the diversion structure was assumed to be still active at the time of data compilation and no endDate was assigned.
no_fill_years	A list of semicolon-separated years where surface water withdrawals were documented to have not occurred.

**Table 4:** Features and descriptions for table of UCOL diversion structures without available time series records.

Table Feature	Feature Description
basin	A three-letter basin code for which subbasin within the UCOL the diversion structure is located: lcr = Little Colorado River Basin, grb = Green River Basin, sjb = San Juan River Basin), cms = Colorado Main Stem (Colorado River and Tributaries outside of the Green, San Juan, and Little Colorado River Basins).
state	A two-letter state code for where the diversion structure is located (az = Arizona, co = Colorado, wy = Wyoming, ut = Utah, nm = New Mexico).
siteName	The site name for each diversion structure within the database that contains state and basin information. Example: grb_ut_vat_diversion
siteUse	The primary water use classification for each diversion structure.
destinationCode	Field specific to the USGS groundwater-surface-water hydrologic model. Values either correspond to model subbasin identification numbers to where water for irrigation is delivered, or model stream segment identification numbers to where water is transferred.
destinationFlag	Field specific to the USGS groundwater-surface-water hydrologic model. A value of −1 denotes water exported from the model area.
dataSource	The data source the Python script accesses for automated retrieval of online records.
cdssID	The CDSS identification number associated with the surface water diversion structure.
adwrID	The ADWR identification number associated with the surface water diversion structure.
adwrCFS	The water right amount in cfs associated with the surface water diversion structure, specific to AZ diversion structures.
decLat	The latitude of the point of diversion associated with each diversion structure.
decLong	The longitude of the point of diversion associated with each diversion structure.
dest_decLat	The latitude of the discrete point of return flows associated with each diversion structure, if applicable
dest_decLong	The longitude of the discrete point of return flows associated with each diversion structure, if applicable
s_t_r	The section, township, and range of the legal point of diversion for WY diversion structures without available time series records.
notes	Notes on the diversion structure, if additional and relevant information was available.

The “input” folder also contains time series withdrawal records that were publicly available, but not available for automated online retrieval. This includes records manually transcribed from commissioner reports and records obtained via data request. These records are grouped by data source; CUWCD records are housed in cuwcd_historical_data, NM OSE records are housed in nmose_historical_data, UTDWR records are housed in utdwr_historical_data, and WY SEO records are housed in wyseo_historical_data. Records are provided as either daily or monthly withdrawal rates in cfs or cfs-days, respectively. Time series records within these folders are stored as CSV files and are named according to structure names (siteName) provided in the Master Table (e.g., “grb_ut_vat_diversion.csv”). Additionally, the “input” folder contains an API token template stored as a text file (“api_token_TEMPLATE.txt”) that can be populated with user credentials to allow for retrieval of records from the CDSS data portal.

The “scripts” folder contains a Python script (ucrb_diversions.py) developed to automate retrieval of daily time series withdrawal records, harmonize these records with other site records that were not available for automated online retrieval, and process all data into a centralized, consistent format. The code utilizes the Master Table and historic time series withdrawal records contained in the “input” folder. Comments and docstrings in the script provide a general summary of data retrieval and processing steps, and a Python environment (“ucrb_diversions.yml”) designed to be compatible with the script is also included.

The “processed” folder contains three tables of daily time series withdrawal records associated with each site stored as CSV files in “wide” format (with date in the leftmost column and site-specific daily withdrawals in cfs in other columns, with column header linking back to the master table via “siteID”). These files are outputs from the code, after automating retrieval of daily time series withdrawal records and combining with all diversion records that were unavailable for automated retrieval (records contained in the “inputs” folder). The first dataset, “combined_diversion_records_raw_cfs.csv,” houses all daily time series records following automated data retrieval and combination with offline records. The second dataset, “combined_diversion_records_filtered_cfs.csv,” is the same dataset, but has been subjected to a filter designed to remove outliers/erroneous values, as detailed in the Methods section. The third dataset, “combined_diversion_records_filtered_cfs_fill_years.csv,” is the final output once years lacking diversion data were filled with estimated values derived from monthly averages, as detailed in the Methods section.

The “processed” folder is provided so that users of the database do not need to run the code in order to obtain the UCOL diversion dataset. However, if desired, users can utilize/modify the code to update time series records, and/or retrieve time series withdrawal records from a customized date range.

## Technical Validation

Since similar site-specific datasets do not exist for areas within the UCOL for direct comparison, these surface water diversion records were utilized to calculate a “natural flows” dataset for 13 USGS streamgages, which was then compared to a natural flows dataset published by USBR^20^ that includes the same locations. Natural flows were calculated as the sum of observed streamflow at the target streamgage and upstream depletions. As withdrawal time series records for diversion structures in this database are records of total withdrawals, and not consumptive use, upstream depletions were estimated by applying consumptive use factors to withdrawal records. A consumptive use factor of 0.6 (e.g., only 60% of diverted water was consumed) was applied to all irrigation diversions. This was estimated to be representative for a mix of flood and sprinkler irrigated lands, which, in Colorado, have been previously estimated to have associated application efficiencies of 30–50% and ~85%, respectively^27^. A consumptive use factor of 0.3 was applied to municipal diversions to account for returns from wastewater and residential irrigation, and is similar to estimates of average system efficiencies for municipal diversions (36%) published in documentation for Colorado Water Resources Planning Models^17^. A consumptive use factor of 1.0 was assigned for interbasin transfers (which export all water out of the UCOL) and industrial diversions, while a consumptive use factor of zero was assigned for non-consumptive hydropower diversions. Water consumption during intrabasin transfers was also assumed to be zero, meaning these diversions are delivered without loss. These methods differ from those utilized in creation of the USBR natural flows dataset. The USBR dataset utilizes estimates of consumptive uses and loses from several operations including irrigated agriculture (derived from original and modified Blaney Criddle methods and crop distribution data), livestock (derived from state agriculture statistics), municipal and industrial operations (estimates reported by hydrologic unit and state), and imports and exports^28^. However, the USBR natural flows dataset also takes into account reservoir operations and losses from reservoir evaporation^28^. We acknowledge the uncertainty in the assumptions associated with consumptive use factors in calculation of our natural flows dataset, and acknowledge that these estimates do not account for reservoir operations within UCOL, but believe these are reasonable basin-wide assumptions to make comparison to the USBR natural flows dataset possible.

Natural flow totals throughout the 1980–2022 study period that were calculated utilizing the UCOL surface water diversion dataset compare well to all available USBR natural flow estimates for applicable USGS streamgages (Table 5). Estimated natural flow values were between 88–124% of USBR natural flow values at all gages (average: 104%, standard deviation: 10%). Though there are uncertainties and assumptions in both natural flow datasets, the close comparison validates data compilation workflow and demonstrates that UCOL surface water use can be represented with site-specific withdrawal records.

**Table 5:** Comparison of USBR natural flows dataset^28^ with the natural flows dataset for this study at applicable USGS streamgage locations^23^.

USGS gage	Site name	percentBOR
09095500	Colorado River Near Cameo, CO	105
09152500	Gunnison River Near Grand Junction, CO	124
09180000	Dolores River Near Cisco, UT	88
09180500	Colorado River Near Cisco, UT	117
09217000	Green River Near Green River, WY	100
09251000	Yampa River Near Maybell, CO	102
09260000	Little Snake River Near Lily, CO	99
09302000	Duchesne River Near Randlett, UT	97
09306500	White River Near Watson, UT	112
09315000	Green River at Green River, UT	97
09328500	San Rafael River Near Green River, UT	93
09379500	San Juan River Near Bluff, UT	112
09380000	Colorado River at Lees Ferry, AZ	106
	**average**	**104.0**
	**standard deviation**	**10.1**

PercentBOR is a comparison of the estimated total natural flow at each gauge for the 1980–2022 study period to that of the BOR natural flows dataset for the same period.

## Usage Notes

The UCOL diversions database^11^ can be accessed at 10.5066/P1496VHX.

This database is limited to diversion structures with publicly available location information (either specific point of diversion location [decimal degrees or section/township/range] or map information [e.g., labelled canals]). Therefore, it likely lacks diversion structures with sensitive location information (e.g., certain municipal diversions) for which location information is unavailable. While this database is likely a sufficient representation of major diversions (greater than 10 cfs) within the UCOL, this database may not contain all major diversion structures. All time series withdrawal records published in this database are publicly available from the data sources listed in the Data Compilation section of this manuscript.

Municipal and industrial diversion structures in the UCOL are likely smaller than diversion structures associated with irrigation canals; therefore, while this dataset provides an inventory of major diversion structures (greater than 10 cfs), using this dataset to estimate total municipal and industrial diversions within the UCOL may result in underreported values.

This database is subject to future improvements and updates, including the addition and modification of sites, refinement of water use types, and the expansion of time series records.

As described above, daily time series records represent total withdrawals, and, therefore, are not necessarily records of consumptive use. However, additional information (e.g., irrigation efficiency estimates, consumptive use factors, etc.) can be leveraged to estimate consumptive use for each diversion structure.

## Data Availability

A Python^12^ script was developed to retrieve, process, filter, and harmonize diversion records from state and federal agency web portals. Code is available with the database^11^, at 10.5066/P1496VHX.
