# Author Correction: Database of surface water diversion sites and daily withdrawals for the Upper Colorado River Basin, 1980–2022

**DOI:** 10.1038/s41597-025-04646-0

**Published:** 2025-02-19

**Authors:** Samuel F. Lopez, Jacob E. Knight, Fred D. Tilman, Melissa D. Masbruch, Daniel R. Wise, Casey J. Jones, Matthew P. Miller

**Affiliations:** 1 U.S. Geological Survey, 2329 W Orton Circle, Salt Lake City, UT 84119 USA; 2 U.S. Geological Survey, 520 N. Park Avenue, Tucson, AZ 85719 USA; 3 U.S. Geological Survey, 601 SW 2nd Ave Suite 1950, Portland, OR 97204 USA; 4 U.S. Geological Survey, 720 Gracern Road Suite 129, Columbia, SC 29210 USA; 5 U.S. Geological Survey, 3215 Marine St, Boulder, CO 80303 USA

**Keywords:** Hydrology, Water resources

Correction to: *Scientific Data* 10.1038/s41597-024-04123-0, published online 21 November 2024

In this article, there was an error in a link associated with reference 18. The error in reference 18 was also repeated in the main text in the following sentence, ‘Publicly available, daily diversion records are housed on the Wyoming State Engineer’s Office (WY SEO) Data Portal (http://meas.ose.state.nm.us/)’. The correct link should be https://seoflow.wyo.gov/. The original article has been corrected.

